# Nanopore sequencing for mapping of retrotransposon integration sites in the *Dictyostelium discoideum* genome

**DOI:** 10.17912/micropub.biology.000543

**Published:** 2022-03-18

**Authors:** Emanuel Barth, Johannes Burggraaff, Akash Srivastava, Thomas Winckler

**Affiliations:** 1 Friedrich Schiller University Jena, Bioinformatics Core Facility; 2 Friedrich Schiller University Jena, Chair of RNA Bioinformatics and High Throughput Analysis; 3 Friedrich Schiller University Jena, Institute of Pharmacy, Chair of Pharmaceutical Biology

## Abstract

The unicellular eukaryote *Dictyostelium discoideum* has a gene-dense haploid genome. This configuration presents mobile elements with the particular challenge of replicating without causing excessive damage to the host through insertional mutagenesis or recombination between repetitive sequences. *D. discoideum* harbors an active population of the retrotransposon TRE5-A that integrates in a narrow window of ~50 bp upstream of tRNA genes. We assume that this integration preference was developed to avoid the disruption of protein-coding genes. Therefore, we recently mapped new integrations of a genetically tagged TRE5-A element at tRNA genes using PCR-based enrichment of integration junctions. However, the PCR-based enrichment produced several artificial DNA fusions that prevented the mapping of integration sites in unknown places of the genome. Here, we reanalyzed the previous experiment using nanopore sequencing. We summarize the advantages and limitations of direct genome resequencing for the mapping of mobile element integrations.

**Figure 1. Nanopore sequencing of TRE5-A bsr integration sites.**
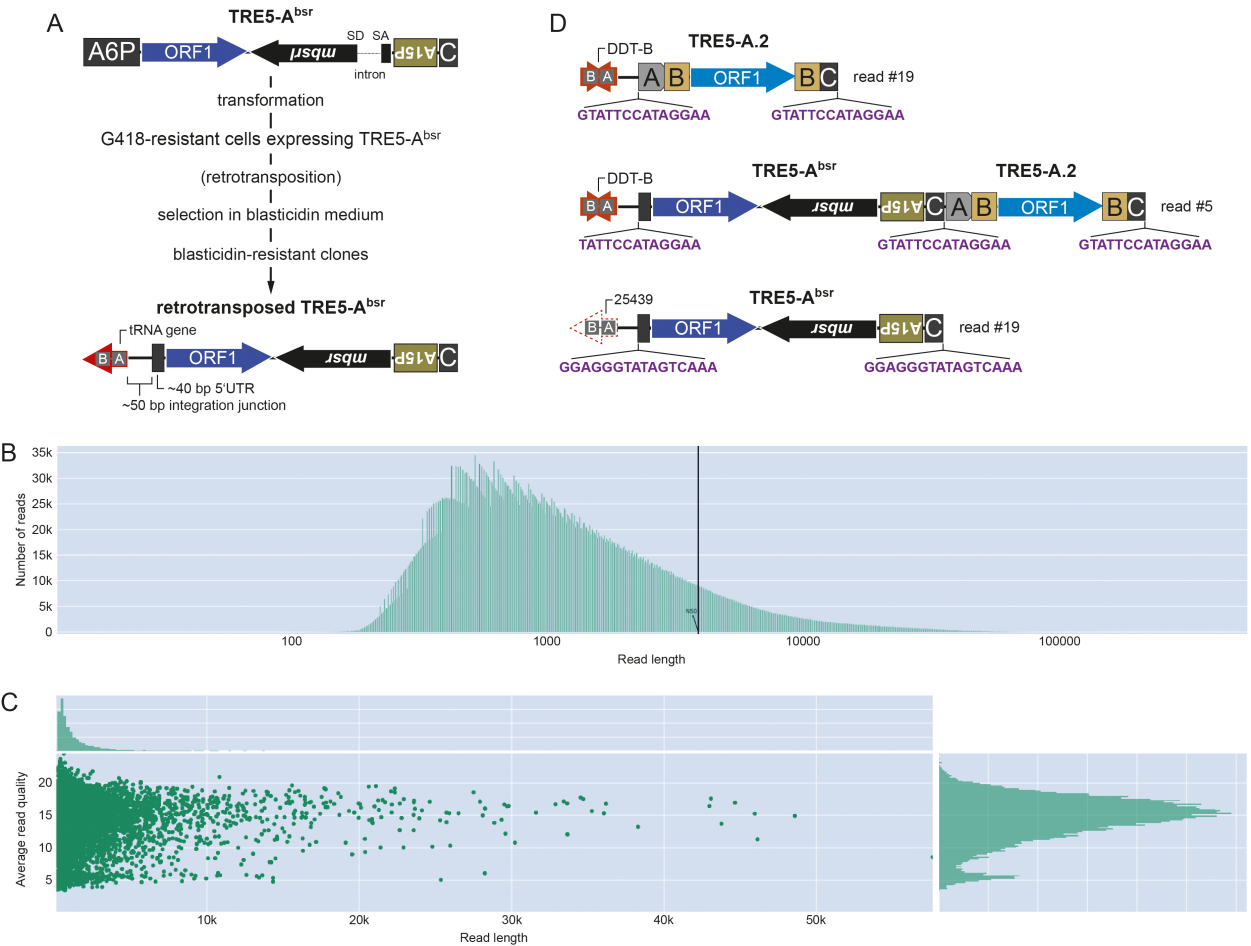
(A) TRE5-A ^bsr^ retrotransposition assay (Siol *et al.* 2011). Expression of the element is driven by the *actin6* promoter (A6P). The element contains a codon-adapted ORF1 sequence and the C-module of TRE5-A. The *mbsrI* gene is inserted in the reverse orientation and is initially nonfunctional due to the presence of the intron. The intron is spliced out of the TRE5-A ^bsr^ RNA after transcription of the element. The *mbsr* gene can now be functionally expressed after reverse transcription and integration of the element. A15P: *actin15* promoter; SD: splice donor; SA: splice acceptor. (B) Log _10_ -transformed length distribution of the nanopore sequenced reads. The black line indicates the N50 length of 3,844 bp. (C) Sequenced read length plotted against the average Phred quality score across reads. The distribution of read length and read quality are shown as histograms on the right and top site, respectively. (D) Examples of full-length TRE5-A ^bsr^ integrations determined by nanopore sequencing. Reads #5 (39,274 bp) and #19 (35,832 bp) partially overlapped and mapped to the distal end of chromosome 5. Both reads indicated a natural TRE5-A.2 element integrated 46 bp upstream of an A/B-box motif (gray boxes) in a rearranged fragment of a DDT-B transposon (nucleotides 428-1+3291-1646 of the 5471 bp DDT-B consensus). Refer to Spaller *et al.* (2017) for further information on the TRE5-A structure. Read #5, but not read #19, showed integration of a TRE5-A ^bsr^ upstream of the A/B-box motif of DDT-B and, therefore, above the already existing TRE5-A.2. Read #19 contained a new TRE5-A ^bsr^ integration 50 bp upstream of the A/B-box motif at position 25439 of the palindromic rDNA element. Purple sequences display target site duplications.

## Description

Transposable elements invade genomes and replicate in host cells with the characteristic of “selfish DNA” (Levin and Devine 2011). Host cells are constantly at risk of losing genome stability due to the amplification of these “genomic parasites” that cause insertional mutagenesis and nonallelic homologous recombination. The risk of insertional mutagenesis seems to be exceptionally high in host cells with gene-dense and haploid genomes and may be more relaxed in organisms with larger diploid genomes (Boeke and Devine 1998). *D. discoideum* is an excellent model to study the mechanisms that transposable elements have developed to amplify in compact genomes to avoid excessive damage (Glöckner *et al* . 2001). Approximately 60% of the *D. discoideum* genome codes for proteins, and intergenic regions are small (Eichinger *et al.* 2005). Therefore, integration into euchromatin regions of the *D. discoideum* genome is only tolerated in the neighborhood of RNA polymerase III genes, in particular tRNA genes, which maintain a certain distance from protein-coding genes (Spaller *et al.* 2016). *D. discoideum* harbors an active population of TRE5-A retrotransposons that are always found in a narrow window of ~50 bp upstream of tRNA genes. Unfortunately, analysis of TRE5-A locations in genome data cannot answer the question of whether the accumulation of the element near tRNA genes is due to an active targeting process to avoid disruption of protein-coding genes or the result of strong selection against cells in which deleterious random integrations have occurred.

We previously followed the replication of a genetically tagged TRE5-A element (TRE5-A ^bsr^ ) and determined integration sites near genes transcribed by RNA polymerase III (Spaller *et al.* 2017) (Figure 1A). TRE5-A ^bsr^ contains the *mbsrI* gene, a selective marker that confers blasticidin resistance to cells in which complete retrotransposition has occurred. In a previous study (Spaller *et al.* 2017), we cultured *D. discoideum* AX2 cells expressing the TRE5-A ^bsr^ element for ~100 generations to allow the retrotransposon to replicate. We then performed blasticidin selection on these cells and collected a pool of ~75,000 blasticidin-resistant clones. Because TRE5-A always integrates in the same orientation upstream of tRNA genes (Figure 1A), we were able to enrich integration junctions of TRE5-A ^bsr^ upstream of tRNA genes by first performing linear amplification-mediated PCR (LAM-PCR) using a TRE5-A ^bsr^ -specific primer, followed by exponential PCR with a TRE5-A ^bsr^ -specific primer and a collection of reverse primers that detect tRNA gene families. Illumina sequencing of pools of purified PCR products allowed us to map TRE5-A ^bsr^ integrations to 384 of the 405 individual tRNA gene loci in the *D. discoideum* genome (Spaller *et al.* 2017). Due to cross-hybridization of some primers with other locations, we unexpectedly discovered TRE5-A ^bsr^ integrations into certain locations on the extrachromosomal palindrome that carries the ribosomal RNA genes. These integration sites mimic tRNA genes because they display canonical A/B-box promoter elements of tRNA genes. Because the ORF1 protein of TRE5-A interacts with subunits of the RNA polymerase III-specific transcription factor TFIIIB in vitro (Chung *et al.* 2007), we hypothesized that TRE5-A integration sites represent locations of active RNA polymerase III transcription.

Using the LAM-PCR approach, we were not able to determine the exact integration specificity of TRE5-A ^bsr^ because we could not isolate integration sites at noncanonical sites with unknown flanking regions. Illumina sequencing produced several artificial DNA fusions that were most likely the results of the triple application of PCR required for the LAM-PCR-based enrichment and subsequent Illumina sequencing of integration junctions. We could not solve this problem by using other, more direct but still PCR-based enrichment protocols such as Vectorette PCR. We assumed that the extreme A+T content of the *D. discoideum* genome was the problem and that a direct genome resequencing approach that does not involve PCR could be helpful. Here, we reanalyzed the previously established pool of blasticidin-resistant clones (Spaller *et al.* 2017) using nanopore sequencing (methodology reviewed in Kono and Arakawa 2019). In this proof-of-concept experiment, we determined that the highly A+T-rich genomic DNA of *D. discoideum* provided a comparable amount and quality of sequencing data to genomes of other organisms. A total of 12,205,887,596 sequenced bases were represented on 6,330,772 reads. The mean read length was 1,928 bp, the N50 value was 3,844 bp, and the longest read covered 533,606 bp. A median Phred quality score Q of 14.9 was achieved, with 87.4% of all sequenced bases having a Q score higher than 10 (Figure 1B, C).

To perform the retrotransposition assay, the TRE5-A ^bsr^ element was first transformed into *D. discoideum* cells, and G418-resistant cells were recovered. These cells were later selected in blasticidin-containing medium to enrich cells with retrotransposition events (Figure 1A). Retrotransposed copies of TRE5-A ^bsr^ can be distinguished from the transformed “master” elements because the intron is removed from the *mbsrI* gene to activate the selective marker ( *mbsr* ). We therefore searched the nanopore sequencing data and identified 124 sequence reads that contained the spliced *mbsr* gene. Of these sequences, 90 reads represented integrations of TRE5-A ^bsr^ at 33 individual tRNA gene loci. In all cases, the integrated TRE5-A ^bsr^ element (~2.4 kb) was complete and flanked by target site duplications of 10-16 bp length. With nanopore sequencing, we determined the first complete sequences of integrated TRE5-A ^bsr^ elements, whereas the LAM-PCR protocol allowed only the analysis of ~100 bp at integration junctions. We further identified 12 reads that confirmed integration of TRE5-A ^bsr^ upstream of known A/B-box positions 22168, 25439 and 26963 of the rDNA palindrome (see Spaller *et al.* 2017 for details). Another 22 reads contained fragments of the TRE5-A ^bsr^ element including the spliced *mbsr* gene that could not be mapped due to missing flanking sequences. Interestingly, we determined a previously unidentified integration site in the putative DNA transposon DDT-B (Figure 1D). This was a specific integration because inspection of the integration site revealed an A/B-box arrangement similar to the A/B-box loci on the rDNA palindrome. Whether this A/B-box motif is related to the replication of DDT-B remains to be determined.

Nanopore sequencing produced ~360-fold coverage. Considering that the sequenced genomic DNA was derived from a collection of ~75,000 blasticidin-resistant clones, i.e., ~75,0000 individual genomes, we could statistically expect to sequence ~200 individual genomes at most. In fact, we identified 124 reads in total that contained new TRE5-A ^bsr^ integrations. Although this result is in good agreement with theoretical considerations, fully saturated mapping of integration sites by nanopore genome resequencing would require much higher coverage. However, this limitation was anticipated and accepted in this particular experiment because we wanted to determine whether nanopore sequencing was capable of predicting whether a particular retrotransposon had the capacity to actively target tRNA genes or integrate randomly. Nanopore sequencing has this power because in this single experiment, we confirmed 33 of 384 (8.5%) previously mapped TRE5-A integration sites at individual tRNA gene loci distributed throughout the genome. Furthermore, we could determine the structure of the entire integrated TRE5-A ^bsr^ element in all integrations as well as sequences of integration junctions and target-site duplications. All these features show that TRE5-A ^bsr^ mimics authentic TRE5-A integrations. In summary, at least in *D. discoideum* , nanopore sequencing is a valuable method for transposon mapping because it avoids the artifacts produced by aberrant PCR-mediated DNA fusion that prevent the identification of unexpected integration sites. In fact, the long nanopore sequencing reads provided interesting additional information not only on the integration behavior of TRE5-A but also on the genome structure itself.

## Methods

The genomic DNA used for nanopore sequencing was the same preparation used for LAM-PCR-based mapping of integrations in a pool of ~75,000 clones of blasticidin-resistant cells selected from a 100-generation culture of cells expressing TRE5-A ^bsr^ (Spaller *et al.* 2017). For nanopore sequencing, 1.8 µg of DNA in 47 µL was used for library preparation with the Oxford Nanopore protocol SQK-LSK109. All steps were followed according to the manufacturer's specifications with increased incubation periods (DNA repair to 15 minutes and adapter ligation to 20 minutes). We also reduced the AMX volume to 3 µl. The final library concentration was 940 ng. The library was then loaded on an R9.4 flow cell and sequenced on a MinION device (ONT). The sequencing run was terminated after 72 h. The raw signal data were base-called using Guppy (v5.0.7). NanoPlot (v1.3.8) was applied to summarize sequencing quality and statistics. Blastn (v2.12.0) (Altschul *et al.* 1990) was used to identify reads containing the spliced *mbsrI* gene. The obtained 124 reads were mapped to the *D. discoideum* reference genome (Eichinger *et al.* 2005) with Minimap2 (v2.24) (Li 2018) using standard parameters.
